# Long-term outcomes after extra-levator versus conventional abdominoperineal excision for low rectal cancer

**DOI:** 10.1186/s12893-022-01692-y

**Published:** 2022-06-22

**Authors:** Haoyu Zhang, Ganbin Li, Ke Cao, Zhiwei Zhai, Guanghui Wei, Chunxiang Ye, Baocheng Zhao, Zhenjun Wang, Jiagang Han

**Affiliations:** Department of General Surgery, Beijing Chaoyang Hosptial, Capital Medical University, No. 8 Gongtinan Lu, Chaoyang District, Beijing, People’s Republic of China

**Keywords:** Extralevator abdominoperineal excision, Abdominoperineal excision, Rectal cancer, Suvival

## Abstract

**Purpose:**

Extralevator (ELAPE) and abdominoperineal excision (APE) are two major surgical approaches for low rectal cancer patients. Although excellent short-term efficacy is achieved in patients undergoing ELAPE, the long-term benefits have not been established. In this study we evaluated the safety, pathological and survival outcomes in rectal cancer patients who underwent ELAPE and APE.

**Methods:**

One hundred fourteen patients were enrolled, including 68 in the ELAPE group and 46 in the APE group at the Beijing Chaoyang Hospital, Capital Medical University from January 2011 to November 2020. The baseline characteristics, overall survival (OS), progression-free survival (PFS), and local recurrence-free survival (LRFS) were calculated and compared between the two groups.

**Results:**

Demographics and tumor stage were comparable between the two groups. The 5-year PFS (67.2% versus 38.6%, log-rank *P* = 0.008) were significantly improved in the ELAPE group compared to the APE group, and the survival advantage was especially reflected in patients with pT3 tumors, positive lymph nodes or even those who have not received neoadjuvant chemoradiotherapy. Multivariate analysis showed that APE was an independent risk factor for OS (hazard ratio 3.000, 95% confidence interval 1.171 to 4.970, *P* = 0.004) and PFS (hazard ratio 2.730, 95% confidence interval 1.506 to 4.984, *P* = 0.001).

**Conclusion:**

Compared with APE, ELAPE improved long-term outcomes for low rectal cancer patients, especially among patients with pT3 tumors, positive lymph nodes or those without neoadjuvant chemoradiotherapy.

**Supplementary Information:**

The online version contains supplementary material available at 10.1186/s12893-022-01692-y.

## Introduction

Since introduction, abdominoperineal excision (APE) has been used as a standard surgical procedure for patients with advanced low rectal cancer [1]. Due to the complex anatomic structure around and in close proximity to the rectum, the “surgical waist” in the tumor-bearing segment around the sphincter complex may cause a positive circumferential resection margin (CRM) and intraoperative perforation (IOP) when removing the levator muscles 2. The positive CRM and IOP rates in the APE group have been reported to be as high as 28.2% and 49%, respectively, resulting in an increased risk of local recurrence and distant metastases [2–4].

In 2007, Holm et al. [5, 6] proposed the concept of extralevator abdominoperineal excision (ELAPE), which fully exposed the perineum and pelvic floor and removed the anal canal and levator muscles to avoid a “surgical waist” [7]. Although some studies have concluded that there were no significant differences in positive CRM and IOP rates between ELAPE and APE, the majority of studies have demonstrated superiority of ELAPE [8, 9]. A further study reported that ELAPE reduces the positive CRM and IOP rates to 20.3% and 8.2%, respectively [10].

ELAPE was developed as a response to poor oncologic outcomes with APE; however, many studies with long-term oncologic outcome data have called into question the value of ELAPE [11–14]. Specifically, a large population-based study from Sweden didn’t demonstrate any survival advantage of ELAPE over APE [12]. The results from these studies [11–14]; however, may be limited by short follow-up periods and small sample sizes. Recently, Shen et al. [6] reported the survival benefit of ELAPE in a long follow-up period compared with APE. Because the superiority of ELAPE when compared to APE is controversial in the recent literature, further studies on this issue are warranted.

Our center began utilizing ELAPE in low rectal cancer patients in 2008 [15]. In the current study, we compared long-term outcomes between ELAPE and APE procedures, and determined the risk factors that affect long-term survival of patients with low rectal cancer.

## Materials and methods

### Patients

This study consisted of patients who underwent ELAPE and APE for low rectal cancer at Beijing Chaoyang Hospital of Capital Medical University between January 2011 and November 2020. During that time period, 1055 patients with advanced rectal cancer underwent surgical resection. Of these patients, 121 consecutive patients with advanced low rectal cancer underwent APE or ELAPE. After exclusions, 114 patients were included in the analysis, including 68 patients who underwent ELAPE and 46 patients who underwent APE. The inclusion criteria were as follows: (1) rectal malignant tumor determined by histology (2) age: 18 to 80 years; (3) ELAPE or APE; (4) Stage II or III determined by preoperative radiographic tests before neoadjuvant therapy; and (5) American Society of Anesthesiologists (ASA) score I and II. The exclusion criteria were as follows: (1) distant metastases found before surgery; (2) acute intestinal obstruction and (3) recurrent cancer. A treatment plan was formulated for each patient by the multi-disciplinary team. Neoadjuvant chemoradiotherapy was recommended for patients with tumors that were concidered to be difficult to achieve R0 resection. The patients received neoadjuvant chemoradiotherapy as a combination of radiation (2.0 Gy/fraction 5 times per week for 5 weeks) and chemotherapy (CapeOX repeated every 3 weeks or mFoLFoX6 repeated every 2 weeks). Surgery was carried out 8 to 12 weeks following neoadjuvant chemoradiotherapy. Patients were completely random in the selection of surgical approaches. The study was conducted in accordance with the Declaration of Helsinki and was approved by the local Ethics Committee of Beijing Chaoyang Hospital. All patients provided their informed consent for the use of their data in the study (2011- ke-143).

### Surgical procedure

The tumor was indicated for abdominoperineal excision if it invaded the levator or anal sphincter or considered to be low for sphincter salvage by surgeons. ELAPE and APE were carried out by two different groups in this department and the patients were randomly assigned to those groups after admission. The surgeons involved in the study have been appropriately trained in ELAPE or APE and both groups had more than ten years experiences in rectal cancer surgery.

The abdominal portion of ELAPE was performed in the spine position. The procedure included mobilization in the mesorectum in the plane outside the mesorectal fascia, and stopped at the top of the coccyx in the back and below the level of the seminal vesicles or cervix anteriorly to achieve a total mesorectal excision. After a colostomy was formed, the patient was rolled over into the prone position for the perineal approach. The perineum continued to be mobilized along the surface of the levator muscle to the pelvic side wall, meeting the abdominal proportion at the start of the levator muscle to entirely remove the levator muscles. The specimens were cylindrical because the levator muscle was still attached to the mesorectum.

APE was performed from the abdominal and perineal portions sequentially in the lithotomy position. The procedure included mobilization in the mesorectum from the levator muscles. When the rectum was fully mobilized, the surgeon moved in between the legs to perform the perineal proportion and the abdominal dissection was performed with excision of the anal canal, including the ischiorectal fat and the lower portions of the levator muscles. There was usually a narrow waist at the lower border of the mesorectum at the level above the levator muscles in the specimen.

### Data collection

The clinicopathologic data, and patients’ status were all obtained from the database. Measurement of the distance between the lower edge of the tumor and the anus was based on a preoperative MRI. The tumor location was determined by radiographic tests, physical examination, or surgical specimens. Adverse events (AEs) during neoadjuvant chemoradiotherapy were reported by the Common Terminology Criteria for Adverse Events version 5.0 (CTCAE) criteria. Positive CRM was defined as cancer cells detected within 1 mm of the resection margin. The clinical TNM staging before neoadjuvant chemoradiotherapy was determined by by preoperative radiographic tests.

Follow‑up evaluations were arranged every 3 months for 2 years and every 6 months thereafter. Chest X‑ray, abdominal CT, and pelvic MRI were performed annually to detect local recurrence or distant metastases. Follow-up evaluations were performed in the outpatient department and by telephone. This study was ended in April 2021.

The following endpoints were estimated: overall survival (OS), defined as the interval from the date of surgery to the date of death from any cause; progression-free survival (PFS), defined as the interval from the date of surgery to the date of first local recurrence, distant metastasis or death; and local recurrence-free survival (LRFS), defined as the interval from the date of surgery to the date of first local recurrence or death. Stratified analyses were performed according to pathological T/N stage, with or without neoadjuvant chemoradiotherapy.

### Statistical analysis

Statistical analysis was performed based on SPSS 25.0 (IBM Corp., Armonk, NY, USA). Independent *t* tests, the Mann-Whitney *U* test, the *chi-square* test and *Fisher’s* exact test were used to compare the statistical differences between the two groups. The Kaplan-Meier curve was used to describe the long-term survival trend. Variables that had a statistically significant association (at *P* < 0.1) with survival of patients on univariate analysis were entered into a multivariable model. Results were reported as number (n) and percentage (%), mean and standard deviation, or hazard ratio (HR) with 95% confidence interval (CI), as appropriate, and were considered statistically significant at *P* < 0.05 in two-tailed tests.

## Results

**Fig. 1:**
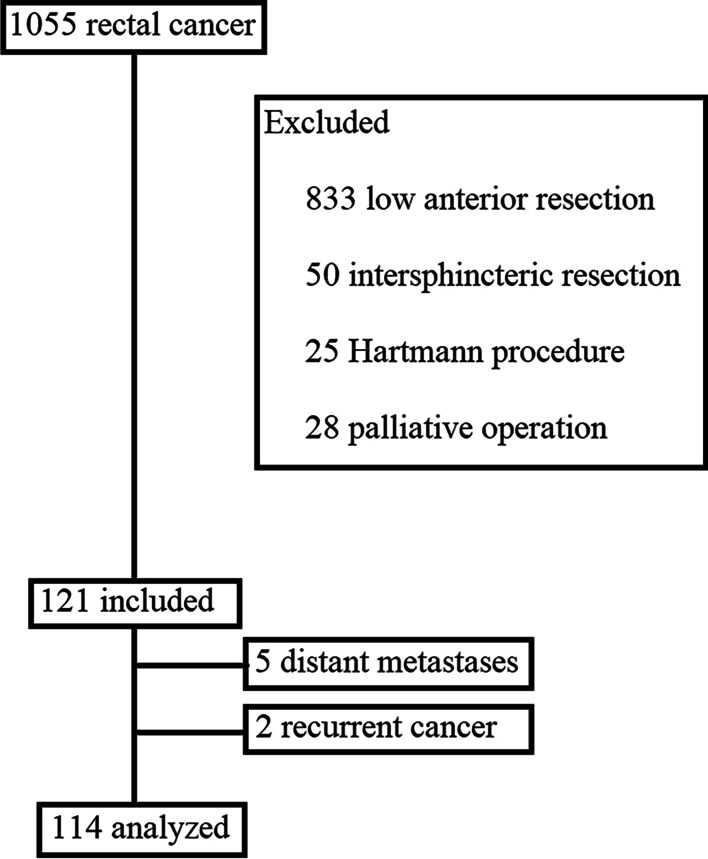
Study flow chart. ELAPE: extralevator abdominoperineal excision; APE: abdominoperineal excision

The median follow-up time was 48.0 months (range 3.0 to 120.0) (Fig. 1). Of the 114 patients, 14 were lost to follow-up before the endpoint occurred, including 8 [10.5% (N = 8)] cases in the ELAPE group and 6 [8.8% (N = 6)] in the APE group; the time from the operation to the most recent follow-up was recorded in the analysis.

### Clinical characteristics

There were no significant differences in gender, age, distance from the anal verge, comorbidities, tumor location, length of postoperative hospitalization, and postoperative complications between the ELAPE and APE groups. Patients who underwent ELAPE were more likely to receive neoadjuvant chemoradiotherapy in this study [45.6% (N = 31) versus 19.6% (N = 9), *P* = 0.004]. The most common AEs during neoadjuvant chemoradiotherapy in the ELAPE group were neutropenia (9.7%), anemia (6.5%) and diarrhea (6.5%). Neutropenia (22.2%) was the only AE in the APE group. There were no significant differences in the grade 3 AEs between the two groups, and no grade 4 or serious adverse events were observed in this study. Compared with the APE group, the ELAPE group had a higher proportion of open surgery [30.9% (N = 21) versus 10.9% (N = 5), *P* = 0.012]. The intraoperative blood loss in the ELAPE group was greater than the APE group (175 versus 100 ml, *P* = 0.004). The total operative time in the ELAPE group were significantly longer than the APE group (318 versus 210 min, *P* < 0.001) (Table 1).

**Table 1:** Comparison of clinical variables of patients between the ELAPE and APE group

	ELAPE (n = 68)	APE (n = 46)	P value
Gender [n (%)]			0.829^a^
Male	43 (63.2)	30 (65.2)	
Female	25 (36.8)	16 (34.8)	
Age (yr, x ± s)	61.3 ± 11.5	64.8 ± 11.1	0.105^d^
BMI (kg/m^2^, x ± s)	24.6 ± 3.7	23.8 ± 3.2	0.669^d^
Cardiovascular disease [n (%)]	23 (33.8)	17 (37.0)	0.731^a^
Diabetes mellitus [n (%)]	10 (14.7)	8 (17.4)	0.700^a^
Cerebral disease [n (%)]	3 (4.4)	2 (4.2)	1.000^a^
Distance from anal verge [cm, M (range)]	3.0 (2.0–3.5)	3.0 (2.0–4.0)	0.315^b^
Clinical T stage [n (%)]			0.593^a^
cT_1 − 2_	8 (11.8)	7(15.2)	
cT_3 − 4_	60 (88.2)	39(84.8)	
Clinical N stage [n (%)]			0.946^a^
cN_0_	38 (55.9)	26(44.1)	
cN_1 − 2_	30 (56.5)	20(43.5)	
Clinical stage [n (%)]			0.717^a^
II	48 (70.6)	31(67.4)	
III	20 (29.4)	15(32.6)	
Neoadjuvant chemoradiotherapy [n (%)]			0.004^a^
Yes	31 (45.6)	9 (19.6)	
No	37 (54.4)	37 (80.4)	
Grade 3/4 adverse events [n (%)]			
Total	8 (25.8)	2 (22.2)	0.827^a^
Neutropenia	3 (9.7)	2 (22.2)	0.668^a^
Anemia	2 (6.5)	0 (0)	1.000^c^
Thrombocytopenia	1 (3.2)	0 (0)	1.000^c^
Diarrhea	2 (6.5)	0 (0)	1.000^c^
Postoperative chemotherapy [n (%)]			0.280^a^
Yes	48 (70.6)	28 (60.9)	
No	20 (29.4)	18 (39.1)	
Approaches of operation [n (%)]			0.012^a^
Laparoscopy assisted	47 (69.1)	41 (89.1)	
Open	21 (30.9)	5 (10.9)	
Total operative time [min, M (range)]	318 (268–360)	210 (180–275)	<0.001^b^
Blood loss [ml, M (range)]	175 (100–200)	100 (80–200)	0.004^b^
Pelvic floor construction [n (%)]			<0.001^a^
Yes	51 (75.0)	2 (4.3)	
No	17 (25.0)	44 (95.7)	
Combined organ resection [n (%)]			1.000 ^c^
Yes	2 (2.9)	1 (2.2)	
No	66 (97.1)	45 (97.8)	
Duration of postoperative hospitalization [d, M(range)]	16 (14–21)	16 (12–26)	0.899^b^
Postoperative drainage time [d, M(range)]	12 (9–14)	8 (6–12)	<0.001^b^
Postoperative complications [n (%)]	19 (27.9)	12 (26.1)	0.827^a^
Abdominal wound healing problem [n (%)]	8 (11.8)	8 (17.4)	0.396^a^
Intestinal obstruction [n (%)]	7 (10.3)	1 (2.2)	0.098^c^
Urinary infection [n (%)]	3 (4.4)	4 (8.7)	0.591^a^
Perineal hernia [n (%)]	3 (4.4%)	1 (2.2%)	0.647^c^

*ELAPE* extralevator abdominoperineal excision, *APE* abdominoperineal excision, *BMI* body mass index; ^a^Chi-square test, ^b^Mann-Whitney *U* test, ^c^*Fisher*’s exact test, ^d^Independent t-tests

### Oncologic characteristics

The tumor characteristics are shown in Table 2. The number of lymph nodes harvested in the ELAPE group was significantly fewer than the APE group (14 versus 16, *P* = 0.034). There were no significant differences between the ELAPE and APE groups in the distance from the lower edge of the tumor to the anus, positive lymph node ratio, clinical T stage, pathologic TN stage, TNM stage, tumor differentiation, lymphovascular invasion, nerve invasion, and positive CRM rate.

**Table 2:** Comparison of oncologic variables of patients between the ELAPE group and APE group

	ELAPE (n = 71)	APE (n = 50)	P value
(y)pT stage [n (%)]			0.836^a^
pT1–2	24 (33.8)	16 (32.0)	
pT3–4	47 (66.2)	34 (68.0)	
ypT stage [n (%)]			1.000^a^
ypT1–2	12 (37.5)	5 (38.5)	
ypT3–4	20 (62.5)	8 (61.5)	
(y)pN stage [n (%)]			0.213^a^
pN0	49 (69.0)	29 (58.0)	
pN1–2	22 (31.0)	21 (42.0)	
ypN stage [n (%)]			1.000^a^
ypN0	23 (71.9)	9 (69.2)	
ypN1–2	9 (28.1)	4 (30.8)	
(y)pTNM stage [n (%)]			0.440^a^
0–I	22 (31.0)	12 (24.0)	
II	27 (38.0)	17 (34.0)	
III	22 (31.0)	21 (42.0)	
Lymph nodes harvested [M (range)]	14 (2–45)	16 (3–42)	0.011^b^
Positive positive lymph node ratio [M (range)]	0 (0–0.11)	0 (0–0.11)	0.895^b^
Histopathology [n (%)]			0.708^a^
Adenocarcinoma	64 (90.1)	44 (88.0)	
Mucinous/signet–ring cell	7 (9.9)	6 (12.0)	
Adenocarcinoma differentiation [n (%)]			0.315^a^
Well and moderate	59 (92.2)	37 (84.1)	
Poor	5 (7.8)	7 (15.9)	
Lymphovascular invasion [n (%)]			0.964^a^
Yes	23 (32.4)	16 (32.0)	
No	48 (67.6)	34 (68.0)	
Nerve invasion [n (%)]			0.177^a^
Yes	15 (21.1)	16 (32.0)	
No	56 (78.9)	34 (68.0)	
Positive CRM [n (%)]	5 (7.0)	10 (22.0)	0.033^a^
Incidence of R0 resection [n (%)]	66 (93.0)	40 (80.0)	0.033^a^

*ELAPE* extralevator abdominoperineal excision, *APE* abdominoperineal excision, *CRM* circumferential resection margin. ^a^Chi-square test, ^b^Mann-Whitney *U* test

### Survival

**Fig. 2:**
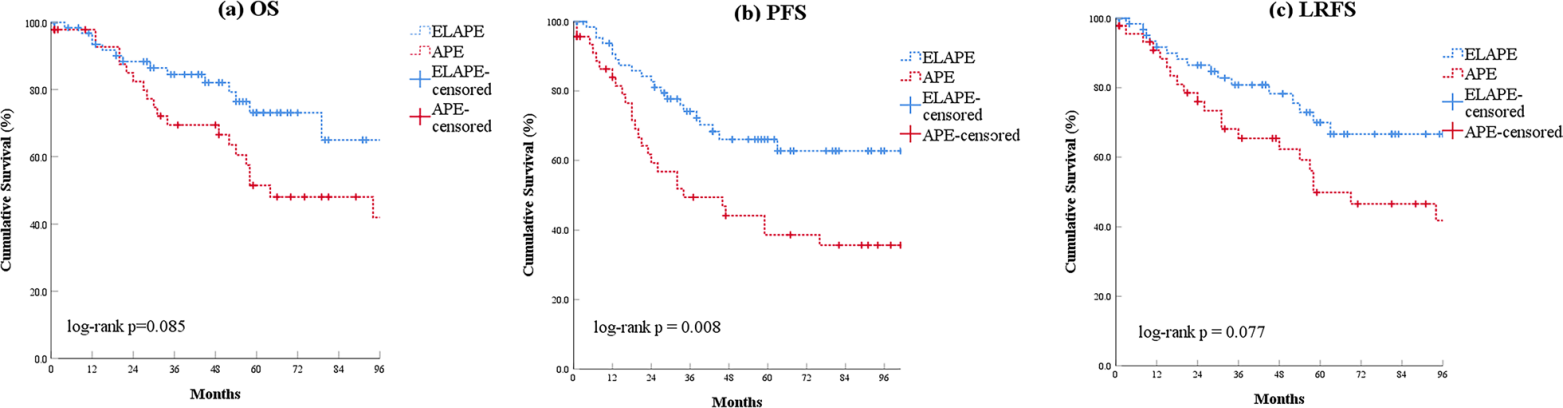
Comparison of overall survival, progression-free survival and local-recurrence-free survival between patients who underwent ELAPE and APE procedures.** a** overall survival; **b** progression-free survival; **c** local recurrence-free survival. ELAPE: extralevator abdominoperineal excision; APE: abdominoperineal excision

Compared with the APE group, patients in the ELAPE group had a longer 5-year PFS (67.2% versus 38.6%; log-rank *P* = 0.008). The 5-year OS and LRFS between the two groups was not statistically different (Fig. 2).

**Fig. 3:**
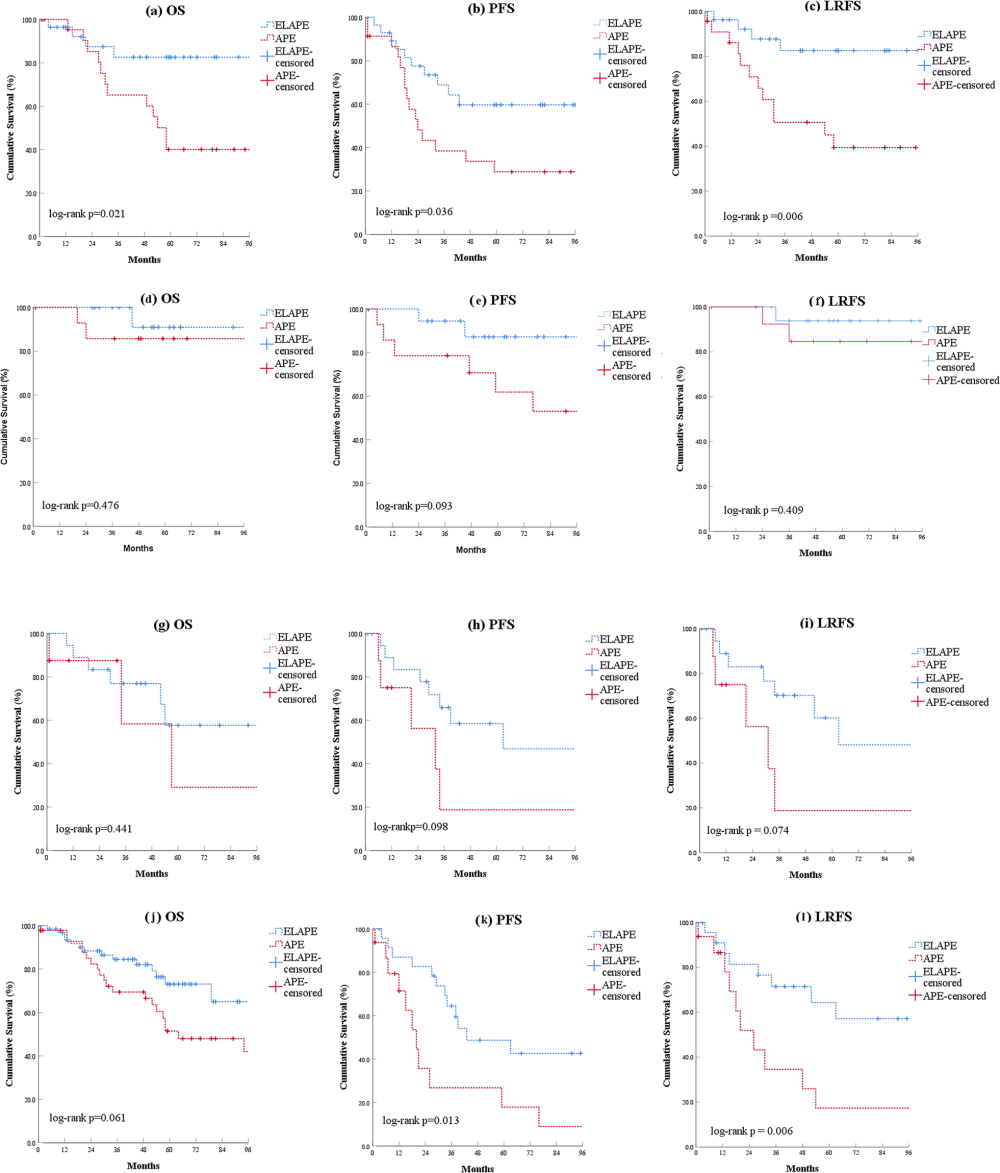
Comparison of overall survival, progression-free survival and local recurrence-free survival between patients of pT_3_, pT_1 − 2_ and pT_4_ tumors or those with positive lymph nodes who underwent ELAPE and APE procedure.** a** pT_3_: overall survival; **b** pT_3_: progression-free survival; **c** pT_3_: local recurrence-free survival; **d** pT_1 − 2_: overall survival; **e** pT_1 − 2_: progression-free survival; **f** pT_1 − 2_: local recurrence-free survival; **g** pT_4_: overall survival; **h** pT_4_: progression-free survival; **i** pT_4_: local recurrence-free survival; **j** positive lymph nodes: overall survival; **(k)** positive lymph nodes: progression-free survival; **l** positive lymph nodes: local recurrence-free survival; ELAPE: extralevator abdominoperineal excision; APE: abdominoperineal excision

Pathologic T_3_ patients in the ELAPE group had a significantly better 5-year OS (82.6% versus 40.1%; log-rank *P* = 0.021), 5-year PFS (59.7 per cent versus 28.8 per cent; log-rank *P* = 0.036), and 5-year LRFS (82.6% versus 39.4%; log-rank *P* = 0.007) than those in the APE group. There were no significant differences in OS, PFS, and LRFS between the two groups of patients with pathologic stages T_0 − 2_ and T_4_. Patients with positive lymph nodes in the ELAPE group had a significantly better 5-year PFS (48.7% versus 17.8%; log-rank *P* = 0.013) and 5-year LRFS (71.5% versus 26.0%; log-rank *P* = 0.006) than the APE group (Fig. 3).

**Fig. 4:**
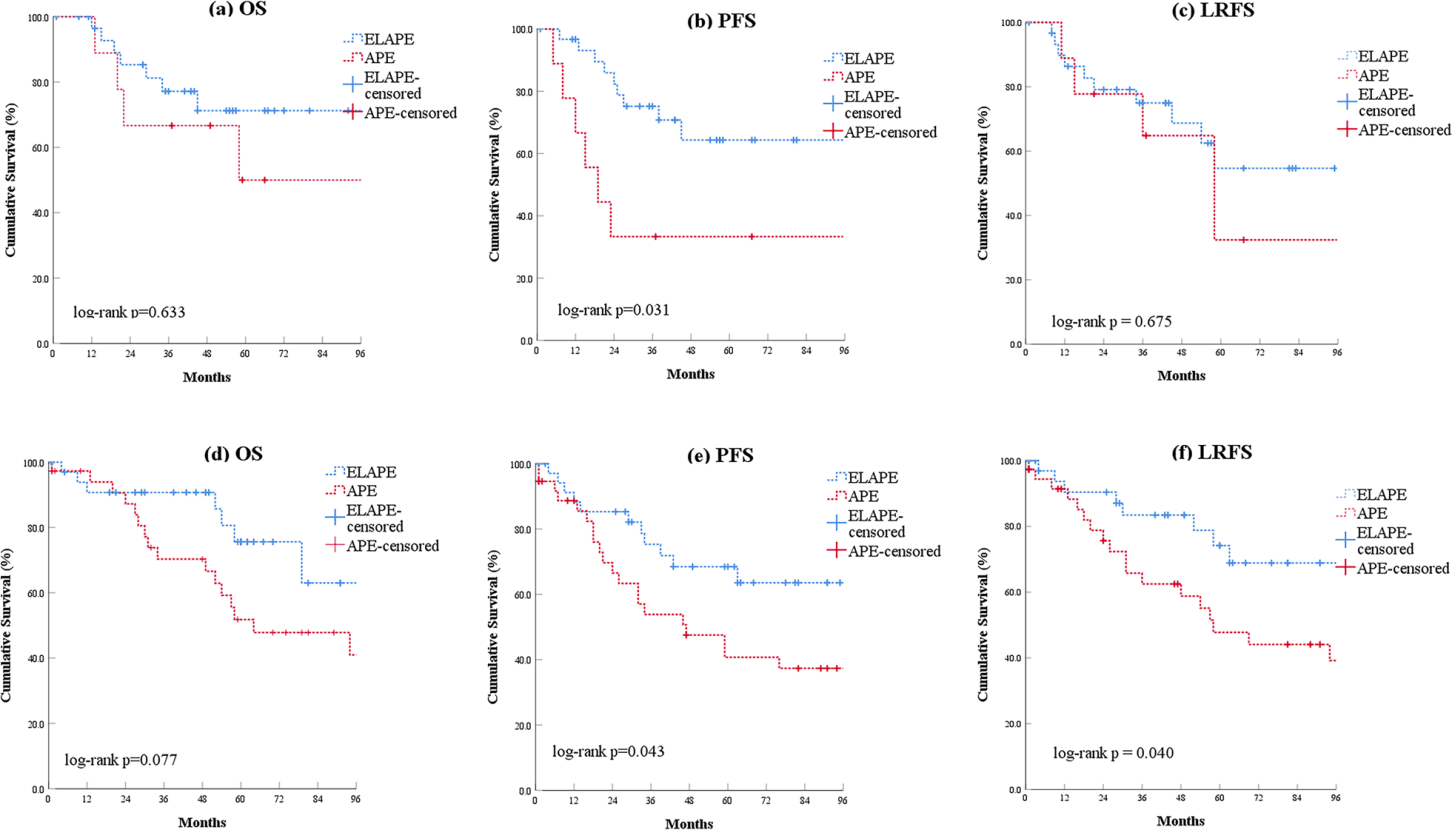
Comparison of overall survival, progression-free survival and local recurrence between patients with or without neoadjuvant chemoradiotherapy who underwent ELAPE and APE procedures. **a** with neoadjuvant chemoradiotherapy: overall survival; **b** with neoadjuvant chemoradiotherapy: progression-free survival; **c** with neoadjuvant chemoradiotherapy: local recurrence-free survival; **d **without neoadjuvant chemoradiotherapy; **e** without neoadjuvant chemoradiotherapy: progression-free survival; **f** without neoadjuvant chemoradiotherapy: local recurrence

For patients with neoadjuvant chemoradiotherapy, ELAPE group showed better 5-year PFS compared to APE group (64.7% versus 33.3%; log-rank *P* = 0.031), but no statistical difference was observed in OS and LRFS between the two groups. Patients without neoadjuvant chemoradiotherapy in the ELAPE group had a significantly better 5-year PFS (68.4% versus 40.7%; log-rank *P* = 0.043) and LRFS (74.2% versus 47.8%; log-rank *P* = 0.040) than those in the APE group (Fig. 4).

### Univariate and multivariate analyses

Univariate analysis showed the following: operation type, pathologic T stage, pathologic N stage, positive lymph node ratio, lymphovascular invasion, nerve invasion, and positive CRM were risk factors for OS; operation type, pathologic T stage, pathologic N stage, positive lymph node ratio, lymphovascular invasion, nerve invasion, and positive CRM were risk factors for PFS; and operation type, pathologic T stage, pathologic N stage, positive lymph node ratio, lymphovascular invasion, nerve invasion, and positive CRM were risk factors for LRFS. Multivariate analysis showed the following: APE HR 3.000, 95% CI 1.171 to 4.970, *P* = 0.004) and advanced pathologic T stage (HR 2.044, 95%CI 1.238 to 3.375, *P* = 0.006) were independent risk factors for OS; APE (HR 2.730, 95%CI 1.506 to 4.984, *P* = 0.001), advanced pathologic N stage (HR 1.865, 95%CI 0.886 to 3.154, *P* = 0.045), and lymphovascular invasion (HR 1.882, 95%CI 1.057 to 3.354, *P* = 0.048) were independent risk factors for PFS; and positive CRM (HR 2.770, 95%CI 1.252–6.130, *P* = 0.012) and advanced pathologic T stage (HR 1.652, 95%CI 1.024–2.665, *P* = 0.040) were independent risk factors for LRFS (Additional file 1: Tables S1–S3).

## Discussion

ELAPE has been performed in patients with low rectal cancer in recent years and has resulted in superior oncologic outcomes compared with APE, but controversy exists regarding the long-term survival of this technique [13, 16]. In the current study long-term outcomes of patients undergoing ELAPE and APE were evaluated, and we showed that ELAPE improved survival of patients with low rectal cancer when compared with APE, especially for patients with pT_3_ tumors, positive lymph nodes or those without neoadjuvant chemoradiotherapy.

Despite a wider range of resection, the number of lymph nodes harvested in the ELAPE group was significantly fewer in number than that in the APE group. Although more tissue was removed with ELAPE, the number of lymph nodes dissected might be not necessarily increased. Alternatively, the effect of a higher proportion of patients receiving neoadjuvant chemoradiotherapy in the ELAPE group. We found that the number of lymph nodes harvested in the patients who received neoadjuvant chemoradiotherapy was significantly fewer than patients who did not in the ELAPE group (11 versus 15, *P* < 0.001). A nationwide study showed that fewer nodes were examined in patients who underwent preoperative chemoradiotherapy compared to patients who did not [17]. Furthermore, although it has been proposed that increasing the number of lymph nodes harvested might increase the probability of recovering positive lymph nodes [18], the number of patients with positive lymph nodes was similar in both groups in the current study. Persiani et al. [19] was also of the opinion that a low number of lymph nodes harvested during surgery after neoadjuvant chemoradiotherapy does not represent inadequate resection or understaging, rather an increased sensitivity to the treatment.

In theory, ELAPE has the potential to reduce local recurrence and improve survival in low rectal cancer patients with more peritumoral tissue removed. The long-term survival of low rectal cancer patients undergoing ELAPE and APE has been a matter of debate in recent years [13, 16, 20]. Klein et al. [13] reported that there is no evidence indicating that ELAPE yields better survival compared to APE. This population, however, had more early-stage tumors (46% considered to be pT_1 − 2_), which might explain why no statistical difference in survival was detected between the two operation types. Shen et al. 6 has proposed a different view. Specifically, a multicenter study revealed that ELAPE was associated with longer survival than APE (median OS, 41.5 versus 29.8 months, *P* = 0.028; median DFS: 38.5 versus 29.3 months, *P* = 0.027; local recurrence rate: 3.80% versus 11.5%, *P* = 0.027). In the current study, we showed that ELAPE improved long-term PFS for all patients with low rectal cancer compared to APE, which was consistent with the results of Shen et al. [6]. Even though a significant OS was not obtained, ELAPE had the added benefit of reducing local recurrence and distant metastases, which facilitated decision-making in selecting the optimal operation type for patients with low rectal cancer.

Further stratified analyses showed patients with pT_3_ tumors had better survival outcomes in the ELAPE group than the APE group with comparable neoadjuvant chemoradiotherapy rate [34.5% (N = 10) versus 21.7% (N = 5), *P* = 0.314]. Due to insufficient resection range at the surgical waist, APE was associated with a higher risk for positive CRM, which could easily lead to local recurrence. Compared to APE, more peritumoral tissues were removed in patients who undergo ELAPE to avoid the formation of a waist at the anorectal junction, thus reducing the positive CRM rate and improving survival outcomes [21]. In the current study, although the difference in positive CRM rate between the two groups was not significant for all patients, the positive CRM rate for pT_3_ tumors in the ELAPE group was significantly lower compared to the APE group [0 versus 13% (N = 3), *P* = 0.045]. In contrast, complete removal of the mesorectum during the ELAPE procedure reduced the perforation rate during the operation and the incidence of local recurrence and metastases [22]. In addition, with less direct manipulation and squeezing of the tumor during ELAPE, the likelihood of distant metastasis caused by the cancer cells entering the blood was reduced. The importance of resection along the lateral fascial plane of the external anal sphincter-levator muscle was emphasized in the ELAPE procedure in compliance with the precise principle of radical removal [23]. Based on our analysis, ELAPE might be more suitable for patients with pT_3_ rectal cancer.

For patients with positive lymph nodes, ELAPE resulted in an incremental survival benefit in the current study with a higher proportion of patients receiving neoadjuvant chemoradiotherapy. For cases that tumor was difficult to remove, neoadjuvant chemoradiotherapy could downstage the tumor to increase the probability of resection [24, 25]. Even though neoadjuvant chemoradiotherapy was essential in the treatment of advanced rectal cancer, we suggested that the ELAPE might be a crucial method by which to promote survival, as confirmed by multivariate analysis in this study. In the current study patients had a significantly higher 5-year PFS in the ELAPE group than the APE group with or without preoperative neoadjuvant chemoradiotherapy. Seshadri et al. [21] also reported that ELAPE resulted in better CRM and IOP outcomes when compared with APE, even after neoadjuvant chemoradiotherapy, but concluded that the operation type still played an important role in long-term survival. Compared with APE, ELAPE removed more tissue to achieve total mesorectal excision, which might have a positive effect on the prognosis of low rectal cancer patients [26].

ELAPE has advantages in treating lower rectal cancer compared to APE, but this superiority wasn’t reflected in pT_0 − 2_ and pT_4_ patients in the current study. For pT_1 − 2_ tumors, in which cancer cells shallowly infiltrate the intestinal wall, ELAPE might not further improve the prognosis with more tissue removed. For pT_4_ tumors, although our study with its relatively small sample size did not demonstrate significant differences in survival between two groups, we thought we couldn’t ignore the effect of location of the tumor and invasion depth on local recurrence. We showed that in pT_4_ tumors the positive CRM were mostly associated the resection margin of the anterior wall. We speculated that located in the anterior wall of the rectum, pT_4_ tumors might invade the prostate or vaginal wall which is associated with local recurrence after resection [27, 28]. Nevertheless, well-designed prospective randomized clinical trials are needed to improve the prognosis of patients with pT_4_ low rectal malignant tumors.

This study had the following limitations. First, the proportion of patients who received neoadjuvant chemoradiotherapy was low, which was 45.1% in ELAPE group and 26.0% in APE group. The low acceptance of neoadjuvant chemoradiotherapy might be associated with patients’ choice and poor general condition (elderly and comorbidities), which might result in increased rate of abdominoperineal resection and poor survival of patients in this study. Second, this was a retrospective study and selection bias was therefore inevitable. The ELAPE group was more likely to receive neoadjuvant chemoradiotherapy in this study. The lower proportion of neoadjuvant chemoradiotherapy in the APE group might contribute to the worse OS and PFS. In this study, stratified analysis by neoadjuvant chemoradiotherapy were performed, and we found that ELAPE might benefit patients with or without neoadjuvant chemoradiotherapy. But to better demonstrate the incremental survival benefit of ELAPE, well-designed, prospective and randomized clinical trials are needed. Finally, the sample size of some subgroups was small and some patients have shorter follow-up, which might affect the results.

## Conclusions

With similar surgical outcomes, ELAPE significantly improved the long-term survival of low rectal cancer compared to APE, especially for patients with pT_3_ and positive lymph nodes. For pT_0 − 2_ and pT_4_ tumors, there was no evidence that ELAPE was superior in improving survival. At present, how to reduce the local recurrence rate and improve the long-term survival of patients with advanced low rectal cancer has not been established. Large-scale, prospective, randomized controlled trials are clearly needed.

## Supplementary Information

**Additional file 1.** Univariate and multivariate analysis for affecting OS, PFS and LRFS in patients with low rectal cancer.

## Data Availability

Data is available from the corresponding author upon reasonable request.
