# Supplementary material for: Long-term outcomes after extra-levator versus conventional abdominoperineal excision for low rectal cancer
Source: BMC Surg. 2022 Jun 22;22:242. doi: 10.1186/s12893-022-01692-y (PMC9219120; doi:10.1186/s12893-022-01692-y)
Supplement: Supplementary file 1 — Additional file 1. Univariate and multivariate analysis for affecting OS, PFS and LRFS in patients with low rectal cancer. [file 12893_2022_1692_MOESM1_ESM.docx]

**Additional file 1**

**Additional file 1:** Table S1. Univariate and multivariate analysis for affecting OS in patients with low rectal cancer.

| Influencing factors [n (%)] | N | Univariate analysis | |  | Multivariate analysis | |
| --- | --- | --- | --- | --- | --- | --- |
|  |  | 5-year OS | P value |  | HR (95% CI) | P value |
| Gender |  |  | 0.312 |  |  |  |
| Male | 73 | 59.8% |  |  |  |  |
| Female | 41 | 58.9% |  |  |  |  |
| Age (years) |  |  | 0.662 |  |  |  |
| > 65 | 55 | 69.2% |  |  |  |  |
| ≤ 65 | 59 | 57.5% |  |  |  |  |
| Distance from anal verge(cm) |  |  | 0.466 |  |  |  |
| > 3 | 38 | 63.6% |  |  |  |  |
| ≤ 3 | 76 | 62.6% |  |  |  |  |
| Comorbidities |  |  | 0.779 |  |  |  |
| Yes | 59 | 68.2% |  |  |  |  |
| No | 55 | 58.3% |  |  |  |  |
| Neoadjuvant chemoradiotherapy |  |  | 0.742 |  |  |  |
| Yes | 40 | 62.1% |  |  |  |  |
| No | 74 | 63.7% |  |  |  |  |
| Postoperative chemotherapy |  |  | 0.303 |  |  |  |
| Yes | 76 | 64.1% |  |  |  |  |
| No | 38 | 60.6% |  |  |  |  |
| Approaches of operation |  |  | 0.952 |  |  |  |
| Laparoscopy assisted | 88 | 65.8% |  |  |  |  |
| Open | 26 | 52.1% |  |  |  |  |
| Operation type |  |  | 0.085* |  |  | 0.004 |
| ELAPE | 68 | 73.1% |  |  | 1.000 |  |
| APE | 46 | 51.4% |  |  | 3.000 (1.171, 4.970) |  |
| Blood loss (ml) |  |  | 0.837 |  |  |  |
| > 100 | 57 | 59.4% |  |  |  |  |
| ≤ 100 | 57 | 67.1% |  |  |  |  |
| Pathological T stage |  |  | 0.011* |  |  | 0.006 |
| T0-2 | 34 | 88.7% |  |  | 1.000 |  |
| T3 | 52 | 55.1% |  |  | 2.044 (1.238, 3.375) |  |
| T4 | 28 | 45.8% |  |  |  |  |
| Pathological N stage |  |  | 0.056* |  |  | 0.062 |
| N0 | 74 | 72.2% |  |  | 1.000 |  |
| N1-2 | 40 | 47.5% |  |  | 2.186 (0.824, 3.743) |  |
| Tumor differentiation |  |  | 0.608 |  |  |  |
| Well and Moderate | 94 | 65.5% |  |  |  |  |
| Poor | 8 | 60.0% |  |  |  |  |
| Mucinous/signet-ring cell | 12 | 48.9% |  |  |  |  |
| Histopathology |  |  | 0.337 |  |  |  |
| Adenocarcinoma | 102 | 65.1% |  |  |  |  |
| Mucinous/signet-ring cell | 12 | 48.9% |  |  |  |  |
| Lymphovascular invasion |  |  | 0.068* |  |  | 0.358 |
| Yes | 36 | 52.2% |  |  | 1.417 (0.673, 2.984) |  |
| No | 78 | 69.6% |  |  | 1.000 |  |
| Nerve invasion |  |  | 0.030 |  |  | 0.656 |
| Yes | 29 | 43.5% |  |  | 1.219 (0.511, 2.908) |  |
| No | 85 | 71.5% |  |  | 1.000 |  |
| Lymph nodes harvested |  |  | 0.298 |  |  |  |
| ≥ 12 | 80 | 57.0% |  |  |  |  |
| ˂ 12 | 34 | 78.2% |  |  |  |  |
| Positive positive lymph node ratio |  |  | 0.094 |  |  |  |
| 0 | 70 | 73.4% |  |  | 1.000 | 0.823 |
| ≤ 0.12 | 10 | 41.7% |  |  | 0.899 (0.352, 2.296) |  |
| 0.12 | 28 | 52.0% |  |  |  |  |
| CRM |  |  | 0.003 |  |  | 0.106 |
| Positive | 14 | 32.2% |  |  | 3.224 (1.340, 7.753) |  |
| Negative | 100 | 68.2% |  |  | 1.000 |  |

OS: overall survival; HR: hazard ratio; 95%CI: 95% confidence interval; ELAPE: extralevator abdominoperineal excision; APE: abdominoperineal excision; BMI: body mass index; CRM: circumferential resection margin.

**Additional file 1:** Table S2. Univariate and multivariate analysis for affecting PFS in patients with low rectal cancer.

| Influencing factors [n (%)] | N | Univariate analysis | |  | Multivariate analysis | |
| --- | --- | --- | --- | --- | --- | --- |
|  |  | 5-year PFS | P value |  | HR (95% CI) | P value |
| Gender |  |  | 0.342 |  |  |  |
| Male | 73 | 50.5% |  |  |  |  |
| Female | 41 | 52.4% |  |  |  |  |
| Age (years) |  |  | 0.104 |  |  |  |
| > 65 | 55 | 48.0% |  |  |  |  |
| ≤ 65 | 59 | 61.0% |  |  |  |  |
| Distance from anal verge (cm) |  |  | 0.463 |  |  |  |
| > 3 | 38 | 56.7% |  |  |  |  |
| ≤ 3 | 76 | 53.8% |  |  |  |  |
| Comorbidities |  |  | 0.999 |  |  |  |
| Yes | 59 | 52.2% |  |  |  |  |
| No | 55 | 57.4% |  |  |  |  |
| Neoadjuvant chemoradiotherapy |  |  | 0.524 |  |  |  |
| Yes | 40 | 54.2% |  |  |  |  |
| No | 74 | 55.4% |  |  |  |  |
| Postoperative chemotherapy |  |  | 0.615 |  |  |  |
| Yes | 76 | 54.1% |  |  |  |  |
| No | 38 | 55.9% |  |  |  |  |
| Approaches of operation |  |  | 0.965 |  |  |  |
| Laparoscopy assisted | 88 | 56.3% |  |  |  |  |
| Open | 26 | 48.9% |  |  |  |  |
| Operation type |  |  | 0.008 |  |  | 0.001 |
| ELAPE | 68 | 67.2% |  |  | 1.000 |  |
| APE | 46 | 38.6% |  |  | 2.730 (1.506, 4.984) |  |
| Blood loss (ml) |  |  | 0.872 |  |  |  |
| > 100 | 57 | 52.6% |  |  |  |  |
| ≤ 100 | 57 | 57.1% |  |  |  |  |
| Pathological T stage |  |  | 0.002 |  |  | 0.091 |
| T0-2 | 34 | 70.6% |  |  | 1.000 |  |
| T3 | 52 | 51.2% |  |  | 1.661 (1.094, 2.522) |  |
| T4 | 28 | 33.8% |  |  |  |  |
| Pathological N stage |  |  | 0.027* |  |  | 0.045 |
| N0 | 74 | 63.7% |  |  | 1.000 |  |
| N1-2 | 40 | 37.8% |  |  | 1.865 (0.886, 3.154) |  |
| Tumor differentiation |  |  | 0.776 |  |  |  |
| Well and Moderate | 94 | 55.6% |  |  |  |  |
| Poor | 8 | 60.0% |  |  |  |  |
| Mucinous/signet-ring cell | 12 | 45.5% |  |  |  |  |
| Histopathology |  |  | 0.489 |  |  |  |
| Adenocarcinoma | 102 | 55.9% |  |  |  |  |
| Mucinous/signet-ring cell | 12 | 45.5% |  |  |  |  |
| Lymphovascular invasion |  |  | 0.013 |  |  | 0.048 |
| Yes | 36 | 39.8% |  |  | 1.882 (1.057, 3.354) |  |
| No | 78 | 63.5% |  |  | 1.000 |  |
| Nerve invasion |  |  | 0.007 |  |  | 0.583 |
| Yes | 29 | 30.3% |  |  | 1.325 (0.650, 2.699) |  |
| No | 85 | 63.3% |  |  | 1.000 |  |
| Lymph nodes harvested |  |  | 0.502 |  |  |  |
| ≥ 12 | 80 | 55.3% |  |  |  |  |
| ˂ 12 | 34 | 64.2% |  |  |  |  |
| Positive positive lymph node ratio |  |  | 0.039 |  |  |  |
| 0 | 70 | 63.6% |  |  |  | 0.969 |
| ≤ 0.12 | 10 | 25.0% |  |  | 1.052 (0.479, 2.311) |  |
| 0.12 | 28 | 43.2% |  |  |  |  |
| CRM |  |  | 0.002 |  |  | 0.249 |
| Positive | 14 | 16.9% |  |  | 3.233 (1.276, 8.144) |  |
| Negative | 100 | 60.7% |  |  | 1.000 |  |

PFS: progression-free survival; HR: hazard ratio; 95%CI: 95% confidence interval; ELAPE: extralevator abdominoperineal excision; APE: abdominoperineal excision; CRM: circumferential resection margin.

**Additional file 1:** Table S3. Univariate and multivariate analysis for affecting LRFS in patients with low rectal cancer.

| Influencing factors [n (%)] | N | Univariate analysis | |  | Multivariate analysis | |
| --- | --- | --- | --- | --- | --- | --- |
|  |  | 5-year LRFS | *P* value |  | HR (95% CI) | *P* value |
| Gender |  |  | 0.498 |  |  |  |
| Male | 73 | 57.4% |  |  |  |  |
| Female | 41 | 58.5% |  |  |  |  |
| Age (years) |  |  | 0.237 |  |  |  |
| > 65 | 55 | 49.9% |  |  |  |  |
| ≤ 65 | 59 | 65.5% |  |  |  |  |
| Distance from anal verge (cm) |  |  | 0.414 |  |  |  |
| > 3 | 38 | 60.8% |  |  |  |  |
| ≤ 3 | 76 | 58.9% |  |  |  |  |
| Comorbidities |  |  | 0.866 |  |  |  |
| Yes | 59 | 59.2% |  |  |  |  |
| No | 55 | 57.9% |  |  |  |  |
| Neoadjuvant chemoradiotherapy |  |  | 0.513 |  |  |  |
| Yes | 40 | 57.6% |  |  |  |  |
| No | 74 | 56.4% |  |  |  |  |
| Postoperative chemotherapy |  |  | 0.727 |  |  |  |
| Yes | 76 | 57.9% |  |  |  |  |
| No | 38 | 56.4% |  |  |  |  |
| Approaches of operation |  |  | 0.824 |  |  |  |
| Laparoscopy assisted | 88 | 57.5% |  |  |  |  |
| Open | 26 | 48.6% |  |  |  |  |
| Operation type |  |  | 0.077 |  |  | 0.087 |
| ELAPE | 68 | 70.0% |  |  | 1.000 |  |
| APE | 46 | 49.8% |  |  | 1.826 (0.917-3.636) |  |
| Blood loss (ml) |  |  | 0.197 |  |  |  |
| > 100 | 57 | 53.9% |  |  |  |  |
| ≤ 100 | 57 | 64.4% |  |  |  |  |
| Pathological T stage |  |  | 0.028 |  |  | 0.040 |
| T0-2 | 34 | 83.5% |  |  | 1.000 |  |
| T3 | 52 | 53.1% |  |  | 1.652 (1.024-2.665) |  |
| T4 | 28 | 45.6% |  |  |  |  |
| Pathological N stage |  |  | 0.018 |  |  | 0.154 |
| N0 | 74 | 71.3% |  |  | 1.000 |  |
| N1-2 | 40 | 47.5% |  |  | 1.713 (0.817-3.592) |  |
| Tumor differentiation |  |  | 0.206 |  |  |  |
| Well and Moderate | 94 | 62.9% |  |  |  |  |
| Poor | 8 | 60.0% |  |  |  |  |
| Mucinous/signet-ring cell | 12 | 45.5% |  |  |  |  |
| Histopathology |  |  | 0.202 |  |  |  |
| Adenocarcinoma | 102 | 60.2% |  |  |  |  |
| Mucinous/signet-ring cell | 12 | 45.5% |  |  |  |  |
| Lymphovascular invasion |  |  | 0.076 |  |  | 0.559 |
| Yes | 36 | 48.6% |  |  | 1.121 (0.602-2.561) |  |
| No | 78 | 68.3% |  |  | 1.000 |  |
| Nerve invasion |  |  | 0.095 |  |  | 0.586 |
| Yes | 29 | 39.8% |  |  | 0.781 (0.321-1.903) |  |
| No | 85 | 66.9% |  |  | 1.000 |  |
| Lymph nodes harvested |  |  | 0.688 |  |  |  |
| ≥ 12 | 80 | 55.7% |  |  |  |  |
| ˂ 12 | 34 | 71.6% |  |  |  |  |
| Positive positive lymph node ratio |  |  | 0.079 |  |  | 0.214 |
| 0 | 70 | 70.4% |  |  | 1.000 |  |
| ≤ 0.12 | 10 | 41.7% |  |  | 0.638 (0.314-1.297) |  |
| 0.12 | 28 | 48.7% |  |  |  |  |
| CRM |  |  | ˂ 0.001 |  |  | 0.012 |
| Positive | 14 | 26.9% |  |  | 2.770 (1.252-6.130) |  |
| Negative | 100 | 66.3% |  |  | 1.000 |  |

LRFS: local recurrence-free survival; HR: hazard ratio; 95%CI: 95% confidence interval; ELAPE: extralevator abdominoperineal excision; APE: abdominoperineal excision; CRM: circumferential resection margin.
